# Effect of pimobendan on mitral annular dynamics and mitral regurgitation in dogs with myxomatous mitral valve disease as determined by cardiac computed tomography

**DOI:** 10.1093/jvimsj/aalag066

**Published:** 2026-04-21

**Authors:** I-Jung Bernard Chi, Brian A Scansen, E Christopher Orton

**Affiliations:** Department of Clinical Sciences, College of Veterinary Medicine and Biomedical Sciences, Colorado State University, 300 W Drake Road, Fort Collins, CO 80525, United States; Department of Clinical Sciences, College of Veterinary Medicine and Biomedical Sciences, Colorado State University, 300 W Drake Road, Fort Collins, CO 80525, United States; Department of Clinical Sciences, College of Veterinary Medicine and Biomedical Sciences, Colorado State University, 300 W Drake Road, Fort Collins, CO 80525, United States

**Keywords:** calcium sensitizer, electrocardiogram-gated computed tomography, phosphodiesterase III inhibitor, structural heart imaging

## Abstract

**Background:**

Pimobendan might affect mitral annular dynamics (MAD) in dogs with myxomatous mitral valve disease (MMVD); in humans, alterations in MAD are known to increase the severity of mitral regurgitation (MR).

**Hypothesis/Objectives:**

To investigate the short-term effect of pimobendan on MAD, severity of MR, and systemic arterial compliance in dogs with MMVD.

**Animals:**

Twenty dogs with newly diagnosed ACVIM stage B2 MMVD.

**Methods:**

Prospective, open-label, longitudinal study. All dogs underwent echocardiographic and cardiac computed tomographic (CCT) examinations before and 2 weeks after oral administration of pimobendan. Changes in MAD, leaflet-to-annulus index, left heart volumes, regurgitant volume/fraction, and aortic distensibility index were evaluated on CCT.

**Results:**

All dogs completed the study without major adverse events. Pimobendan decreased normalized end-systolic mitral annular area (mean difference of 1.38 cm^2^/m^2^; 95% CI, 0.79-1.97 cm^2^/m^2^; *P* < .001), aortoparietal distance (mean difference of 1.03 mm/kg^1/3^; 95% CI, 0.54-1.53 mm/kg^1/3^; *P* < .001), and intercommissural distance (mean difference of 0.75 mm/kg^1/3^; 95% CI, 0.44-1.06 mm/kg^1/3^; *P* < .001). Mean leaflet-to-annulus index increased (mean difference of 0.06; 95% CI, 0.02-0.09; *P* = .002). Changes in left ventricular end-diastolic and end-systolic volumes, left atrial end-diastolic and end-systolic volumes, regurgitant volume (mean reduction of 0.31 mL/kg; 95% CI, 0.13-0.5 mL/kg), and regurgitant fraction (mean reduction of 9.8%; 95% CI, 5.2%-14.3%) were detected (all *P* < .05) with administration of pimobendan. There was no detectable difference in aortic distensibility index (*P* = .6).

**Conclusions and clinical importance:**

Short-term pimobendan administration decreases MR severity by augmenting systolic contraction of the mitral annulus. The study provides new insight into the mechanism of action of pimobendan in dogs with MMVD.

## Introduction

Pimobendan, an inodilator acting through phosphodiesterase-III inhibition and calcium sensitization, is part of the guideline-directed medical management of myxomatous mitral valve disease (MMVD) in dogs. Pimobendan prolongs survival and time to onset of congestive heart failure in dogs with subclinical MMVD and left heart enlargement (American College of Veterinary Internal Medicine [ACVIM] Stage B2).^1^ Reduction in left heart size and severity of mitral regurgitation (MR) after administration of pimobendan occur in dogs with subclinical MMVD.^2^^,^^3^ In dogs with congestive heart failure stabilized with furosemide monotherapy, pimobendan decreases left heart size and enhances systolic function.^4^ Administration of pimobendan promotes left ventricular (LV) forward stroke volume (FSV) through afterload reduction (systemic vasodilation) and positive inotropy.^3^^,^^5^ The benefits of pimobendan might go beyond these effects.

The geometry and size of the mitral annulus (MA) are critical to the biomechanical function of the MV and can become abnormal in dogs with MMVD.^6^^,^^7^ Moreover, the MA is not a rigid, organic structure but rather a dynamic, functional component of the mitral apparatus. The capacity of the MA to change its shape and size throughout the cardiac cycle is defined as mitral annular dynamics (MAD). Abnormal MAD occur in humans with degenerative MR where the MA undergoes expansion instead of constriction during systole.^8–11^ This is associated with a dynamic increase in regurgitant orifice area throughout ventricular systole.^8–12^ Given the known association between MR severity and MAD, the potential influence of pimobendan on MAD is of relevance in understanding the pharmacodynamic benefit of this drug in dogs with MMVD.

The advent of cardiac-gating and multidetector computed tomography (CT) has made it feasible to capture cardiac motion, quantify cardiac chambers, and characterize the conformational changes of cardiac structures at different cardiac phases with excellent spatial and temporal resolution. These features make cardiac computed tomography (CCT) a useful imaging modality for cardiac volume quantification and characterization of MAD, aided by state-of-the-art imaging software. The objective of this study was to investigate the short-term effects of pimobendan on MAD, MR severity, left ventricular volumes, and aortic distensibility index (a surrogate of systemic arterial compliance) in dogs with newly diagnosed ACVIM stage B2 MMVD using CCT. We hypothesized that pimobendan decreases the severity of MR by enhancing left ventricular contractile function and through systolic contraction of the MA.

## Animals, materials, and methods

### Animals and study design

Client-owned dogs with newly diagnosed ACVIM stage B2 MMVD were prospectively enrolled in this single group, non-blinded, comparison to baseline longitudinal study. All dogs were required to be larger than 4 kg in body weight to ensure optimal spatial resolution of the CCT. The dogs were required to be otherwise healthy and to not be receiving pimobendan or other medications with known cardiovascular effect at the time of enrollment. Once the diagnosis of ACVIM stage B2 MMVD was confirmed during an outpatient appointment or internal cardiac consultation, the option to enroll the dog into the study was offered to the client and the study consent form was signed. At the first study visit, physical examination, complete blood count, diagnostic chemistry profile, and thoracic radiographs were performed to confirm the absence of major comorbidities. A comprehensive transthoracic echocardiographic exam and CCT were acquired under brief sedation. The dog was then discharged with pimobendan (Vetmedin, Boehringer Ingelheim Animal Health USA Inc., Duluth, GA, USA) to be given orally at 0.5-0.6 mg/kg/day divided into twice-daily doses. All dogs returned 14 days after the first visit for a repeat echocardiogram and CCT under the same sedation and imaging protocols (described below). Verbal confirmation was obtained from the owners to ensure the medication had been given consistently over the 14-day period. At the end of the second visit, all dogs were discharged to the owner and maintained on chronic pimobendan therapy at the same dosage. The study protocol was approved by the Institutional Animal Care and Use Committee of Colorado State University (protocol number 1612).

### Echocardiography

Standard 2-dimensional and Doppler echocardiography (Epiq CVx, Philips Healthcare, Andover, MA, USA) was performed at both study visits (before and after initiation of pimobendan therapy) by a cardiology resident (IJBC) supervised by a board-certified cardiologist (BAS). All dogs received butorphanol 0.1-0.3 mg/kg IV for the echocardiographic exam. The diagnosis of ACVIM stage B2 MMVD was made according to the ACVIM consensus guidelines for the diagnosis and treatment of MMVD in dogs.^13^ Adequate LV systolic function, absence of significant semilunar valve disease or other structural abnormalities were confirmed in all dogs. Special attention was paid to ensure the absence of hemodynamically substantial tricuspid regurgitation or right heart enlargement^14^ despite the presence of tricuspid valve degeneration. An estimate of arterial elastance (Ea) was acquired as previously described in another veterinary study.^15^ Echocardiographic Ea was calculated as mean oscillometric blood pressure (obtained during CCT acquisition as described below) divided by the LV FSV (aortic velocity-time integral × aortic valve cross-sectional area) indexed to body weight in kilograms.

### Cardiac computed tomography—image acquisition

A dual source, 384-slice (192 slices in each detector row) CT scanner (SOMATOM Force, Siemens Healthineers USA) was used to acquire all CCT studies. All CCTs were acquired under brief sedation and spontaneous respiration. The sedatives were administered (butorphanol 0.2-0.5 mg/kg IV with titrated boluses of alfaxalone 0.5-1 mg/kg IV) and sedation monitored by the Anesthesia and Pain Management Service at the authors’ institution. The dogs were positioned on the CT table in ventral recumbency with flow-by oxygen supplementation. Pulse rate, peripheral oxygen saturation, and oscillometric blood pressure were monitored during the CCT. Oscillometric blood pressure (ePM 12 M Vet, Mindray Animal Medical, Shenzhen, China) was measured with the inflatable cuff placed on one of the forelimbs and repeated every 2-3 min throughout the sedation. The mean blood pressure recorded at the time of contrast injection was recorded for estimation of systemic arterial compliance in each dog.

A pre-contrast thoracic CT was first acquired and reconstructed to select the monitoring slice for bolus tracking. The region of interest with a threshold of 150 Hounsfield units was placed in the aortic arch to trigger retrospective acquisition of the levophase, and then the dextrophase after an 8- to 12-s delay based on the patient’s heart rate. A triphasic contrast (iohexol 300 mgI/mL, Omnipaque, GE Healthcare) protocol delivering a total of 1.5 mL/kg contrast was used: 1 mL/kg of 100% contrast at 1-3 mL/s (0.21 ± 0.07 mL/kg/s), followed by 1 mL/kg of 50:50 contrast:saline mixture at 1-3 mL/s (0.21 ± 0.07 mL/kg/s), and a final 1 mL/kg saline bolus at 1-3 mL/s. All studies were reconstructed at 0.75 mm slice thickness with the field-of-view containing the entire heart and great vessels. Both the levophase and dextrophase scans were reconstructed into 20 phases at 5% increments of the R-to-R cycle length and stored for further analysis.

### Cardiac computed tomography—image analysis

**Figure 1:**
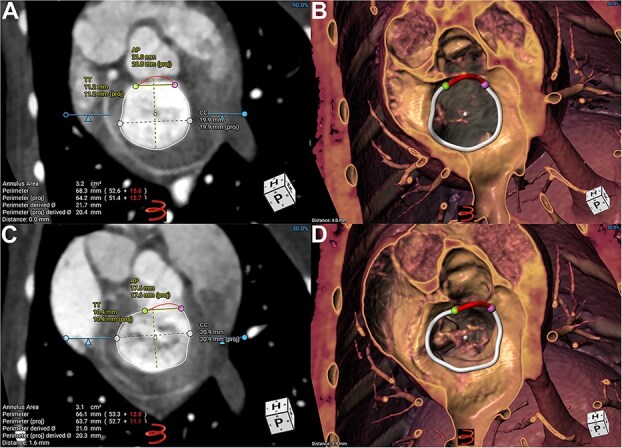
Segmentation and measurements of mitral annular geometry by cardiac computed tomography. Segmented virtual MA on multiplanar reformatting (A and C) and volume rendered (B and D) images from the atrial perspective at end diastole (A and B) and end systole (C and D). Markers were placed at the left (green) and right (pink) fibrous trigones. The mitral annulus was separated into the aortic (red) and parietal (white) portions. Abbreviations: AP = aortoparietal distance; CC = intercommissural distance; MA = mitral annulus; TT = trigone-to-trigone distance.

The MA was manually segmented using dedicated CCT imaging software (3mensio Structural Heart, Pie Medical Imaging, the Netherlands). A total of 16 markers were placed around the MA in multiplanar reformatting to generate a 3-dimensional (3D), saddle-shaped MA ring (Figure 1). The left/lateral and right/medial fibrous trigones were manually identified. After successful segmentation of the MA, the following geometrical measurements were auto-generated (Figure 1): mitral annular area (MAA; defined as the projected area of the segmented MA ring), aortoparietal distance (APD; the linear measurement drawn between the aortic and parietal horns of the saddle across the center of the MA), intercommissural distance (ICD; the maximal linear dimension of the MA orthogonal to the APD), and trigone-to-trigone distance (TTD; the linear dimension between the 2 fibrous trigones). In addition to the automated measurements, the sphericity index (SI) of the MA was calculated as APD/ICD. Leaflet-to-annular index (LAI) was calculated as a measure of MV leaflet coaptation reserve described in previous human and veterinary literature.^16–18^ The lengths of the aortic and parietal MV leaflets corresponding to the middle of the A2 and P2 segments were measured across the center of the MV and summated as the total leaflet length (TLL). The LAI was defined as TLL/APD.

Volumetric measurements from the CCT studies were made with an offline imaging software (IntelliSpace Portal, Philips Healthcare USA). Left ventricular end-diastolic (LVEDV) and end-systolic volumes (LVESV) were measured to quantify LV total stroke volume (TSV) and ejection fraction (EF). Right ventricular TSV was used as a surrogate for LV FSV in the absence of relevant right-sided valvular regurgitation. As a result, the regurgitant volume (RVol) across the MV was estimated by the difference between the left and right ventricular TSVs. Regurgitant fraction (RF) was calculated as RVol/LVTSV. Left atrial end-diastolic (LAEDV, minimal) and end-systolic volumes (LAESV, maximal) were measured with exclusion of the left atrial appendage and pulmonary veins. All linear, area, and volumetric measurements were indexed to (body weight in kilograms)^1/3^, body surface area (in meters squared), or body weight (in kilograms), respectively.

**Figure 2:**
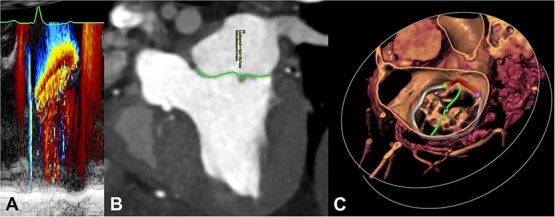
Measuring LAI on cardiac computed tomography. The LAI was measured during the late-systolic phase which is the phase assumed to have the peak effective regurgitant orifice area of mitral regurgitation. (A) Color M-mode across the flow convergence of a mitral regurgitant jet on transthoracic echocardiogram from one of the dogs in the study. The radius of the flow convergence reaches its maximum during the latter half of ventricular systole. (B) Measuring leaflet lengths (green curved lines) and aortoparietal distance (straight white dotted line) by cardiac computed tomography in multiplanar reformatting at the late-systolic phase. (C) The same LAI measurements in B on a volume rendered image of the mitral valve (left atrial perspective). The mitral annulus was segmented as described in Figure 1. Abbreviation: LAI = leaflet-to-annulus index.

Measurements of MA geometry and the cardiac volumes were made at different phases of the cardiac cycle: mid-diastole (the beginning of diastasis), late-diastole (just before atrial contraction), end-diastole (after atrial contraction and before onset of ventricular systole), mid-systole (at first phase of aortic valve opening), and end-systole (at minimal LV volume). In addition, a late-systolic phase between mid- and end-systole was selected to measure the LAI (Figure 2).

Systemic arterial compliance was estimated by the aortic distensibility index (ADI).^19^^,^^20^ The ADI was defined as the fractional cross-sectional area change in the ascending aorta (at the middle point between the sinotubular junction of the aortic valve and the origin of the brachycephalic trunk) divided by systemic pulse pressure (oscillometric systolic arterial pressure—diastolic arterial pressure).

The 2 sets (pre- and post-pimobendan) of CCT studies were analyzed at separate timepoints by a single observer. The observer was blinded from the results of the pre-pimobendan measurements when analyzing the post-pimobendan studies.

### Statistics

All continuous variables were examined for normality using Shapiro–Wilk test and normal quantile-quantile plot. Continuous variables are summarized as mean ± SD or median with interquartile range for parametric and nonparametric sample data, respectively. Mean pre- and post-pimobendan differences for the studied variables were calculated and reported with 95% CIs. Paired *t*-test or Wilcoxon signed-rank test was used to assess whether paired differences in measurements are centered around zero, depending on the normality of the differences in measurements. The significance level (α) of the study was set at 0.05. Statistical computing and graphics were compiled using an open-source programming language and software environment (R version 4.4.1 [Race for your life]; RStudio version 2024.09.0 + 375).

## Results

Twenty client-owned dogs (Table 1) of various breeds were enrolled at Colorado State University Veterinary Teaching Hospital between February 2022 and September 2023. All dogs had unremarkable blood work and no major extracardiac abnormalities on baseline thoracic radiographs. The mean dosage of pimobendan was 0.57 mg/kg/day, orally, and ranged from 0.5 to 0.64 mg/kg/day. All dogs received pimobendan, orally, twice daily for the entire study period and completed the study without discontinuation of medication or major adverse events (only 1 dog experienced mild, self-limiting diarrhea). Dogs did not develop signs of congestive heart failure or overt disease progression during the study period.

**Table 1:** Demographics of the study sample presented as mean (range of data).

	**All dogs (*n* = 20)**
**Age (years)**	10.38 (6-14)
**Sex**	Male 13, female 7
**Body weight (kg)**	9.84 (4.3-19.6)
**LVIDdN**	1.84 (1.7-2.05)
**LA/Ao**	1.83 (1.61-2.5)
**Pimobendan (mg/kg/day)**	0.57 (0.5-0.64)
**Breed**	Border Collie (1), Boston Terrier (1), Cavalier King Charles Spaniels (4), Chihuahua (1), Havanese (2), Mixed breed (10), Yorkshire Terrier (1)

Abbreviations: LA/Ao = left atrium-to-aortic dimension ratio in short axis; LVIDdN = normalized left ventricular internal dimension in diastole.

### The effect of pimobendan on mitral annular dynamics

**Figure 3:**
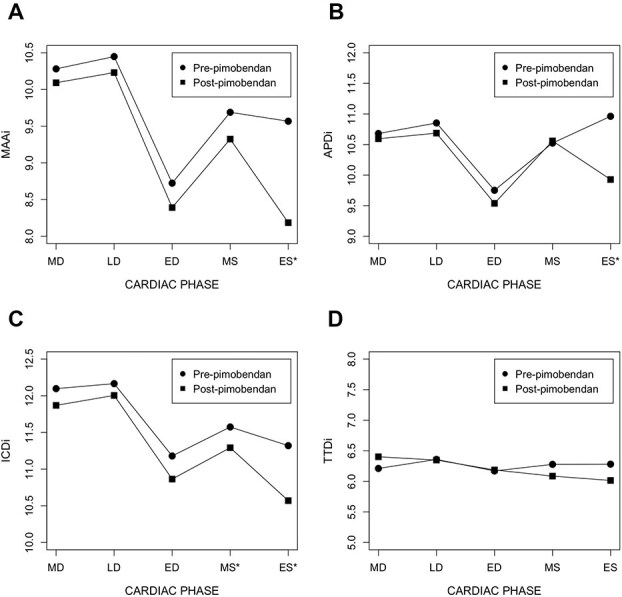
Mitral annular dynamics before and after pimobendan in dogs with ACVIM stage B2 myxomatous mitral valve disease. Temporal changes in mitral annular area (A), aortoparietal distance (B), intercommissural distance (C), and trigone-to-trigone distance (D) before and after pimobendan are compared at each cardiac phase. Linear measurements were indexed to (body weight in kilograms)^1/3^ and the mitral annular area was indexed to body surface area in square meters. *Significant difference in measurement after pimobendan (*P* < .05). Abbreviations: ACVIM = American College of Veterinary Internal Medicine; APDi = indexed aortoparietal distance; ED = end diastole; ES = end systole; ICDi = indexed intercommissural distance; LD = late diastole; MAAi = indexed mitral annular area; MD = mid diastole; MS = mid systole; TTDi = indexed trigone-to-trigone distance.

At baseline, the MA reached its minimal size at end-diastole (after atrial contraction) and then underwent systolic expansion (Figure 3). This pattern was observed in MAA and ICD but was most prominent in APD where the end-systolic dimension exceeded the diastolic dimension (Figure 3B). The TTD had no detectable differences throughout the cardiac cycle (Figure 3D). Short-term administration of pimobendan decreased the mean end-systolic

**Figure 4:**
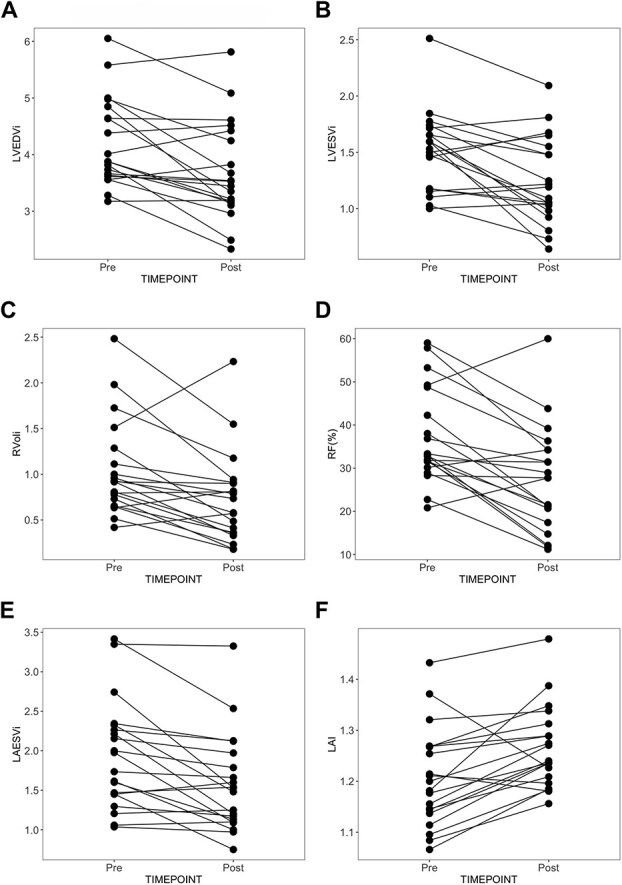
Changes in indexed left ventricular volume in diastole (A) and systole (B), severity of mitral regurgitation by regurgitant volume (C) and regurgitant fraction (D), left atrial volume (E), and leaflet-to-annulus index (F) after short-term administration of pimobendan. Volumetric measurements are indexed to bodyweight in kilograms. All measurements decreased after administration of pimobendan (*P* < .05). Abbreviations: LAI = leaflet-to-annulus index; LAESVi = indexed left atrial end systolic volume; LVEDVi = indexed left ventricular end-diastolic volume; LVESVi = indexed left ventricular end-systolic volume; RVoli = indexed regurgitant volume; RV(%) = regurgitant fraction.

MAA (mean difference of 1.38 cm^2^/m^2^; 95% CI, 0.79-1.97 cm^2^/m^2^; *P* < .001) and APD (mean difference of 1.03 mm/kg^1/3^; 95% CI, 0.54-1.53 mm/kg^1/3^; *P* < .001) whereas the mean ICD was reduced at both mid-systole (mean difference of 0.28 mm/kg^1/3^; 95% CI, 0.01-0.56 mm/kg^1/3^; *P* = .04) and end-systole (mean difference of 0.75 mm/kg^1/3^; 95% CI, 0.44-1.06 mm/kg^1/3^; *P* < .001). There were no detectable statistical differences in mean end-diastolic MAA (*P* = .06), APD (*P* = .2), and ICD (*P* = .07). No statistical differences were noted for mean TTD or SI at any cardiac phase. The mean LAI increased (mean difference of 0.06; 95% CI, 0.02-0.09; *P* = .002) due to reduction in APD at late systole (*P* = .004) with no detectable change in TLL (*P* = .6) (Figure 4F). Paired statistics of MAD and LAI changes are summarized in Tables 2 and 3.

**Table 2:** Geometrical measurements of the mitral annulus before and after pimobendan at different cardiac phases with p values representing change pre- to post-pimobendan therapy.

	**Time**	**Cardiac phase**														
		**MD**			**LD**			**ED**			**MS**			**ES**		
		**Mean ± SD**	**Mean difference, 95% CI**	***P* value**	**Mean ± SD**	**Mean difference, 95% CI**	***P* value**	**Mean ± SD**	**Mean difference, 95% CI**	***P* value**	**Mean ± SD**	**Mean difference, 95% CI**	***P* value**	**Mean ± SD**	**Mean difference, 95% CI**	***P* value**
**MAA**	Pre	10.28 ± 1.39	0.19, −0.26-0.64	.4	10.45 ± 1.47	0.22, −0.28-0.72	.4	8.72 ± 1.43	0.33, −0.02-0.69	.06	9.69 ± 1.81	0.37, −0.15-0.88	.2	9.56 ± 2.08	1.38, 0.79-1.97	<.001^a^
	Post	10.09 ± 1.61			10.23 ± 1.78			8.39 ± 1.26			9.32 ± 1.97			8.19 ± 1.67		
**APD**	Pre	10.68 ± 0.93	0.09, −0.29-0.46	.6	10.85 ± 0.99	0.17, −0.24-0.57	.4	9.75 ± 0.93	0.21, −0.12-0.55	.2	10.52 ± 1.39	−0.04, −0.45-0.38	.9	10.96 ± 1.49	1.03, 0.54-1.53	<.001^a^
	Post	10.59 ± 1.23			10.69 ± 1.30			9.54 ± 0.99			10.56 ± 1.43			9.93 ± 1.30		
**ICD**	Pre	12.10 ± 0.94	0.23, −0.15-0.6	.2	12.17 ± 0.87	0.16, −0.17-0.49	.3	11.18 ± 0.97	0.31, −0.03-0.65	.07	11.57 ± 0.96	0.28, 0.01-0.56	.04^a^	11.32 ± 1.19	0.75, 0.44-1.06	<.001^a^
	Post	11.87 ± 0.94			12.00 ± 1.10			10.86 ± 0.99			11.29 ± 1.08			10.57 ± 0.99		
**TTD**	Pre	6.21 ± 0.58	−0.19, −0.41-0.03	.09	6.36 ± 0.53	0.01, −0.23-0.25	.9	6.17 ± 0.56	0.02, −0.20-0.17	.9	6.28 ± 0.61	0.19, −0.02-0.4	.07	6.28 ± 0.78	0.27, −0.002-0.5	.05
	Post	6.40 ± 0.61			6.35 ± 0.65			6.19 ± 0.64			6.09 ± 0.67			6.02 ± 0.64		
**SI**	Pre	0.88 ± 0.07	−0.01, −0.05-0.03	.6	0.89 ± 0.07	0.002, −0.03-0.04	.9	0.87 ± 0.06	−0.01, −0.04-0.03	.7	0.91 ± 0.07	−0.03, −0.06-0.004	.08	0.97 ± 0.08	0.03, −0.003-0.06	.08
	Post	0.89 ± 0.09			0.89 ± 0.09			0.88 ± 0.08			0.94 ± 0.09			0.94 ± 0.09		

^a^ *P* values reaching statistical significance (*P* < .05).

Abbreviations: APD = aortoparietal distance; ED = end diastole; ES = end systole; ICD = intercommissural distance; LD = late diastole; MAA = mitral annular area; MD = mid diastole; MS = mid systole; SI = sphericity index; TTD = trigone-to-trigone distance.

All linear and area measurements were indexed to (body weight in kilograms)^1/3^ and body surface area (in meters squared), respectively.

**Table 3:** Comparisons of left heart size, severity of mitral regurgitation, and systemic blood pressure and arterial compliance before and after administration of pimobendan.

	**Time**	**Mean ± SD or median, IQR**	**Mean difference (95% CI)**	***P* value**
**LAI**	Pre	1.21 ± 0.10	−0.06 (−0.09, −0.02)	.002^a^
	Post	1.26 ± 0.08		
**LVEDV (mL/kg)**	Pre	4.21 ± 0.80	0.52 (0.22, 0.81)	.002^a^
	Post	3.69 ± 0.86		
**LVESV (mL/kg)**	Pre	1.52 ± 0.35	0.28 (0.12, 0.44)	.002^a^
	Post	1.24 ± 0.38		
**LVEF (%)**	Pre	64.01 ± 5.24	−2.43 (−5.82, 0.96)	.1
	Post	66.44 ± 6.92		
**LVSV (mL/kg)**	Pre	2.7 ± 0.58	0.24 (0.01, 0.48)	.04^a^
	Post	2.45 ± 0.61		
**RVol (mL/kg)**	Pre	1.04 ± 0.52	0.31 (0.12, 0.5)	.003^a^
	Post	0.73 ± 0.50		
**RF (%)**	Pre	37.1 ± 11.04	9.75 (5.16-14.34)	<.001^a^
	Post	27.35 ± 12.22		
**LAESV (mL/kg)**	Pre	1.94 ± 0.69	0.37 (0.17, 0.57)	<.001^a^
	Post	1.51, 1.125 - 1.833		
**LAEDV (mL/kg)**	Pre	0.96 ± 0.30	0.18 (0.08, 0.28)	.001^a^
	Post	0.78 ± 0.24		
**Ea (mmHg/mL/kg)**	Pre	64.55 ± 18.94	3.74 (−4.7, 12.18)	.4
	Post	60.81 ± 20.01		
**ADI (mmHg^−1^ × 10^3^)**	Pre	5.379, 4.456 - 6.616	N/A	.6
	Post	5.15 ± 1.48		
**MBP (mmHg)**	Pre	89.50 ± 22.76	−3.75 (−15.27, 7.77)	.5
	Post	93.25 ± 11.82		

^a^ *P* values reaching statistical significance (*P* < .05).

Abbreviations: ADI = aortic distensibility index; Ea = arterial elastance; LAEDV = left atrial end diastolic volume; LAESV = left atrial end systolic volume; LAI = leaflet-to-annulus index; LVEDV = left ventricular end diastolic volume; LVEF = left ventricular ejection fraction; LVESV = left ventricular end systolic volume; LVSV = left ventricular stroke volume; MBP = mean blood pressure; RF = regurgitant fraction; RVol = regurgitant volume.

### The effect of pimobendan on mitral regurgitation and left heart volumes

There was a reduction in mean LVEDV (mean difference of 0.52 mL/kg; 95% CI, 0.22-0.81 mL/kg; *P* = .002) by 12.4% and LVESV (mean difference of 0.28 mL/kg; 95% CI, 0.12-0.44 mL/kg; *P* = .002) by 18.4% (Figure 4A and B) after pimobendan. Mean LV TSV reduced (mean difference of 0.24 mL/kg; 95% CI, 0.01-0.48 mL/kg; *P* = .04) while no statistical difference could be detected in mean LVEF (*P* = .1). Both the mean RVol (mean difference of 0.31 mL/kg; 95% CI, 0.12-0.5 mL/kg; *P* = .003) and RF (mean difference of 9.75%; 95% CI, 5.16%-14.34%; *P* < .001) decreased by approximately 30% (Figure 4C and D). Left atrial volume at both end-diastole (mean difference of 0.18 mL/kg; 95% CI, 0.08-0.28 mL/kg; *P* = .001) and end-systole (mean difference of 0.37 mL/kg; 95% CI, 0.17-0.57 mL/kg; *P* < .001) reduced by 9.4% and 22.2% (Figure 4E), respectively. Paired statistics of changes in volumetric measurements are summarized in Table 3.

### The effect of pimobendan on estimates of systemic arterial compliance/elastance

There were no detectable differences in either the echocardiographic Ea (*P* = .4) nor CCT-derived ADI (*P* = .6) after pimobendan therapy. The study sample also failed to demonstrate a difference in mean blood pressure (*P* = .5) between the 2 visits (Table 3).

## Discussion

This study found that short-term pimobendan administration augmented systolic contraction of the MA, likely contributing to decreased MR severity, thereby resulting in a pharmacologic annuloplasty and limiting the size of the regurgitant orifice. Similar observations have been made in humans where dobutamine infusion reduced the mitral annular diameter and severity of MR without concurrent systemic vasodilation.^21^ Previous animal studies have also found an association between LV systolic function and the regurgitant orifice area of experimentally induced MR.^22^^,^^23^

The MV is a highly dynamic structure with complex anatomy and biomechanics. Rather than an organic anatomic structure, the canine MA can be considered a “virtual” structure consisting of the aortomitral fibrous continuity (the aortic portion) and the atrioventricular myocardial conjunction of the LA and LV (the parietal portion).^24^ Due to the fibrous and fixed nature of the trigones, the TTD of the MA does not undergo relevant change throughout the cardiac cycle (Figure 3). Rather, the parietal portion of the MA changes its size and shape with relaxation and contraction of the LA and LV myocardium. The dynamics of the MA play an important role in the biomechanical function of the MV. An experimental canine study showed that the shape and size of the MA in healthy dogs are influenced by both atrial and ventricular contractions throughout the cardiac cycle.^25^ During atrial contraction, contraction of the MA occurs substantially to facilitate leaflet coaptation and closure right before the onset of ventricular systole. The MA will continue to contract in ventricular systole to further optimize MV coaptation and reduce the amount of mechanical stress on the leaflets and subvalvular apparatus. The normal MAD are affected by changes in LA or LV contractile function, loading conditions of the left heart, or the presence of MR.^25^

All dogs in this study showed abnormal MAD characterized by systolic expansion instead of contraction of the MA, especially in the aortoparietal dimension. This specific pattern of abnormal MAD is shared by humans with degenerative MV disease as described in multiple studies.^8–12^ The timing of the paradoxical increase in MA dimension is coincident with the rapid expansion of LA volume secondary to MR, which can further worsen malcoaptation of the MV leaflets and MR during ventricular systole.^26^ This could partially explain the late-systolic peaking in regurgitant orifice area of MR that has been observed in humans,^27–30^ and might occur as well in dogs. This study demonstrates that pimobendan rectifies systolic expansion of the MA, which mitigates the functional/dynamic expansion of the regurgitant orifice area (resulting in a so-called pharmacologic annuloplasty).^21^ The increase in late-systolic LAI can also be considered indirect evidence of this phenomenon where a smaller annular dimension is now covered by the same amount of TLL as existed at baseline. This effect is independent of the absolute size of the MA as there was no detectable difference in the mean diastolic dimensions of the MA after receiving pimobendan despite the reduction in mean LVEDV and LAEDV.

Segmenting and tracking a dynamic 3D structure like the MA can be challenging for cardiac imaging modalities. Previous veterinary studies have utilized real-time 3D transthoracic and transesophageal echocardiography to determine MA morphology in dogs but were limited to a single-phase analysis in systole.^6^^,^^7^^,^^31^ While echocardiography provides high-resolution imaging of the valve leaflets, cross-sectional imaging such as CCT provides a wider field of view including the entire left heart and all functional components of the mitral apparatus. The use of CCT is reported in many veterinary studies but none have utilized the technology to study the MV apparatus in dogs with MMVD. This study demonstrates that detailed geometrical assessment of the canine MA at multiple cardiac phases is feasible with the use of CCT. Future studies are needed to explore other applications of CCT in canine MMVD such as perioperative imaging for planning and evaluating surgical/transcatheter interventions.

The capability to quantify cardiac chamber volumes also affords CCT a role in the quantification of MR severity. The volumetric quantification method used to obtain RVol and RF in this study was adopted from previous studies in humans with MMVD.^32^ With isolated MR, the difference in TSV between the left and right ventricles should equal to the RVol, assuming the FSV is the same out of the pulmonary and aortic outflow tracts. With the technical challenges in performing cardiac magnetic resonance imaging for MR quantification in dogs,^33^ quantification of RVol using CCT could be considered as a viable alternative.

There were no detectable changes in mean blood pressure, echocardiographic Ea, or CCT ADI. As a result, decreased arterial impedance through increased large artery compliance or decreased arterial resistance did not appear to be an important mechanism for MR reduction after pimobendan therapy in these dogs. Notably, it is equally possible that these estimates of arterial distensibility and vascular resistance are insensitive, confounded by inaccuracy of oscillometric blood pressure, or undetectable due to small sample size and inadequate statistical power.

There are a few limitations relevant to this study. First, this was designed as a paired single-arm study because withholding pimobendan in dogs with stage B2 MMVD would be ethically challenging. Without an untreated placebo group, we cannot fully separate treatment effect from disease or measurement variability. However, each dog served as its own control, and the short follow-up interval reduces the likelihood that the observed changes reflect natural variation alone. Second, the study only represents the short-term effect of pimobendan administration. Future studies of longer duration would be required to understand if the pharmacological effect persists to later stages of the disease. Third, the small sample size can underpower the statistical analyses performed in this study. Fourth, the indirect estimations of systemic arterial compliance in the study were not validated by any form of reference method and the mean blood pressure measurements were not acquired simultaneously to the echocardiographic examination which might introduce physiologic variability into the calculation of Ea. Oscillometric blood pressure measurement in the dog is also known to have inaccurate or imprecise readings.^34^ Last, the study only focuses on the dynamics of the MA and does not address other components of the mitral apparatus such as valve leaflets, chordae tendineae, or papillary muscles. It is possible that pimobendan also affects MR severity through other biomechanical influences on these structures.

This study demonstrated that in a small group of dogs with ACVIM stage B2 MMVD, pimobendan augments systolic contraction of the MA, and reverses the detrimental end-systolic expansion that occurs with primary MR. We postulate that this mechanical benefit, or pharmacologic annuloplasty, helps to explain the clinical benefit and reduction in heart size seen in prior studies.^1^^,^^3^ Greater understanding of the mechanistic benefit of pimobendan could facilitate refinement of the clinical indication for this therapy in dogs, while also having relevance to management of degenerative MV disease in other species.

## Off-label antimicrobial declaration

The authors declare no off-label use of antimicrobials.

## Institutional animal care and use committee or other approval declaration

Approved by the Institutional Animal Care and Use Committee at Colorado State University (protocol number 1612).

## Abbreviations

3D: 3-dimensional
ACVIM: American College of Veterinary Internal Medicine
ADI: aortic distensibility index
APD: aortoparietal distance
CCT: cardiac computed tomography
Ea: arterial elastance
FSV: forward stroke volume
LAEDV: left atrial end diastolic volume
LAESV: left atrial end systolic volume
LV: left ventricle
LVEDV: left ventricular end diastolic volume
LVESV: left ventricular end systolic volume
MA: mitral annulus
MAA: mitral annular area
MAD: mitral annular dynamics
MMVD: myxomatous mitral valve disease
MR: mitral regurgitation
MV: mitral valve
SI: sphericity index
TSV: total stroke volume
TTD: trigone-to-trigone distance
